# Association of Bisphenol A Exposure with LINE-1 Hydroxymethylation in Human Semen

**DOI:** 10.3390/ijerph15081770

**Published:** 2018-08-17

**Authors:** Youping Tian, Xiaoyu Zhou, Maohua Miao, De-kun Li, Ziliang Wang, Runsheng Li, Hong Liang, Wei Yuan

**Affiliations:** 1 Key Laboratory of Reproduction Regulation of NPFPC, SIPPR, IRD, Fudan University, Shanghai 200237, China; 16211150003@fudan.edu.cn (Y.T.); zouxy2001@163.com (X.Z.); miaomaohua@163.com (M.M.); wangziliang1986@126.com (Z.W.); yuanwei11@yahoo.com (W.Y.); 2 School of Public Health, Fudan University, Shanghai 200032, China; 3 Division of Research, Kaiser Foundation Research Institute, Kaiser Permanente Northern California, Oakland, CA 94612, USA; de-kun.li@kp.org

**Keywords:** Bisphenol A, sperm, DNA hydroxymethylation, LINE-1, 5hmC

## Abstract

Bisphenol A (BPA), an exogenous endocrine-disrupting chemical, has been shown to alter DNA methylation. However, little information is available about the effect of BPA exposure on DNA hydroxymethylation in humans. The objective of the present study was to examine whether BPA exposure was associated with DNA hydroxymethylation in human semen samples. We measured urine BPA levels and LINE-1 hydroxymethylation in 158 male factory workers selected from an occupational cohort study conducted in China between 2004 and 2008. Among them, there were 72 male workers with occupational BPA exposure (BPA-exposed group) and 86 male workers without occupational BPA exposure (unexposed group). Multivariate linear regression models were used to examine the association of exposure to BPA with LINE-1 hydroxymethylation. LINE-1 was more highly hydroxymethylated in the BPA-exposed group than in the unexposed group (median 12.97% vs. 9.68%, respectively; *p* < 0.05), after adjusting for the potential confounders. The medians of 5-hydroxymethylcytosine (5hmC) generally increased with increasing urine BPA levels: 8.79%, 12.16%, 11.53%, and 13.45%, for undetected BPA and corresponding tertiles for the detected BPA, respectively. After analysis using data at individual level, our findings indicated that BPA exposure was associated with alterations of sperm LINE-1 hydroxymethylation, which might have implications for understanding the mechanisms underlying BPA-induced adverse effects on male reproductive function.

## 1. Introduction

Bisphenol A (BPA), an exogenous endocrine-disrupting chemical, is extensively used in the production of polycarbonate plastic and epoxy resins [1] and is ubiquitously present in the environment, owing to the wide use of consumer products in daily life. In the United States, the Centers for Disease Control and Prevention (CDC) detected BPA in 95% of urine samples in the general population using isotope dilution gas chromatography-mass spectrometry (GC-MS) [2]. In vitro studies have shown that BPA can interact with estrogen receptors α and β, leading to estrogenic effects [3], and act as an androgen receptor antagonist with strong anti-androgenic effects [4]. The endocrine-disrupting effects of BPA on male reproductive health have raised concerns over the past decades and a number of animal studies have shown that low environmental levels of BPA exposure can cause adverse effects on male reproduction [5,6]. In humans, several studies have demonstrated associations between BPA and semen quality parameters or reproductive hormones [7,8,9]. Our previous studies also found inverse associations between occupational BPA exposure and semen quality or male sexual function [10,11]. However, the molecular mechanism of BPA toxicity on human reproduction remains unclear.

Epigenetic features are highly sensitive to environmental exposures, and therefore can function as important biomarkers for the effects of environmental exposures [12,13]. The best-characterized epigenetic mark in sperm is DNA 5-methylcytosine (5mC) modification within CpG dinucleotides, which has been shown to be altered following BPA exposure [14,15]. A growing body of evidence suggests that BPA-induced alterations in DNA methylation might be an important intermediate in the pathway of BPA exposure and the impairment of male reproductive functions [15,16]. DNA methylation is involved in regulating promoter activity and is very dynamic since 5mc could be converted to 5-hydroxymethylcytosine (5hmC), through consecutive oxidation reactions catalyzed by ten-eleven-translocation (TET) protein family members [17,18,19]. Given that 5hmC is mainly present at actively transcribed genes [20,21], and that it can bind chromatin regulator proteins, such as MeCP2 [22] and MBD3 [23], some researchers propose that 5hmC is a predominantly stable DNA modification distinct from 5mC and, therefore, is likely to have a different regulatory function rather than existing merely as a transient species [24]. The enrichment of 5hmC is potentially crucial for regulation of gene expression during spermatogenesis [25]. While two recent animal studies suggested that exposure to BPA inhibited global DNA hydroxymethylation or TETs-mediated DNA demethylation [26,27], little information is available about the effect of BPA exposure on global 5hmC in humans.

Long interspersed element-1 (LINE-1), comprising of approximately 17% of human genomic DNA [28], is the most abundant and the only active autonomous non-LTR retrotransposon in the human genome. Genomic studies have revealed that 5hmC is enriched in intergenic regions, such as LINE-1, satellites, and gene bodies of synaptic plasticity-related loci [29,30]. Gan et al. found that 5hmC is mainly located in long interspersed element sequences (LINEs) in pachytene spermatocytes (pacSC, one type of spermatogenic cells) [25]. Moreover, our recent genome-wide alteration study (GWAS) in DNA hydroxymethylation found that BPA could raise 5hmC rate in four sperm genes (AChE, ATP2A1, PPP2R3C, and LINE-1), using the pooled sperm samples [31]. In the present study, we examined the association between exposure to BPA and LINE-1 hydroxymethylation in individual semen samples (un-pooled analysis).

## 2. Materials and Methods

### 2.1. Study Population and Data Collection

In the present study, we included 158 male factory workers. The workers were selected from an occupational cohort study examining the health effects of occupational BPA exposure in several regions of China between 2004 and 2008, as previously described [10,11,14]. In brief, male workers (BPA-exposed group) were recruited from manufacturers of epoxy resin, who used BPA as one of their raw materials. We confirmed that they were exposed to BPA in the workplace via both spot and personal air sampling measurements. Unexposed workers were identified and recruited from factories which had no known BPA exposure and were not exposed to known reproductive toxicants in the workplace in the same region during the same time period. The control factories (e.g., construction material manufacturers, water supply factories, machinery factories and garment factories) were from the same jurisdiction of the health department overseeing the occupational health of the participating BPA-exposed factory. This study was performed by three academic and research institutions and was approved by the research ethics committees of all of them (ethical permit number: PJ2015-16). All participants gave written informed consent before participation in the study.

Among 514 recruited male workers, we selected two regions (Yueyang and Wuxi, *N* = 395) for LINE-1 hydroxymethylation assay because of limited funding. Those who (1) were aged between 20 to 45 years and (2) have semen specimens available for the assay of hydroxymethylation and urine sample for assay of BPA were recruited to the present study. A total of 158 male workers were finally included in the present analysis, including 72 in BPA-exposed group and 86 in unexposed group. Through an in-person interview, all participants were asked to fill out a detailed questionnaire, including information on demographic characteristics, lifestyle factors (i.e., smoking and alcohol consumption) and history of diseases (i.e., acute or chronic disease of liver, kidney or other organs, diagnosed by a physician). In addition, they were asked to provide urine and semen specimens.

### 2.2. BPA Measurement

A detailed description of BPA measurement can be found elsewhere [10,11,14]. Briefly, two urine samples, pre-shift and post-shift, were collected from each male worker in the BPA-exposed group (varied between 7AM to 12PM depending on the schedule of shifts), and mean BPA concentrations of pre- and post-shift urine BPA of each worker were calculated and used to represent the actual BPA exposure levels for the statistical analyses. Only one spot urine sample was collected from workers in the BPA-unexposed group (varied between 8AM to 4PM) because their work shift should not have effects on their urine BPA levels. The total urinary concentrations of BPA (free plus conjugated species) were measured using high-performance liquid chromatography (HPLC), as described in a previous study [32]. We mixed urine samples with phosphorous acid buffer and β-glucuronidase (Sigma Chemical Co, St. Louis, MO, USA) for hydrolyzation, and then were extracted twice with ether (HPLC grade; Dikma, Lake Forest, CA, USA). The supernatants were evaporated with nitrogen gas and the residue was dissolved in 60% acetonitrile and analyzed by HPLC with fluorescence detection. The limit of detection (LOD) for BPA concentrations was 0.31 mg/L, which was calculated with the method recommended by EPA [32], and was comparable to LODs reported in previous studies [1]. To account for urinary dilution, urinary BPA concentrations were divided by urinary creatinine concentrations and reported in micrograms per gram creatinine.

### 2.3. Semen Collection

The collection and examination of all semen specimens strictly followed the standards set by the World Health Organization [33]. Participants were asked to abstain from sexual activity for at least 2 days, but not exceeding 7 days, before the specimen collection. In a private room with a temperature of 20–28 °C a clean and wide-mouthed container was utilized to collect semen specimens through masturbation and ejaculation. We performed macroscopic (e.g., liquefaction, appearance, viscosity, volume and pH) and microscopic analyses (e.g., concentration, motility, vitality and morphology) according to the WHO manual [33]. All semen analyses were conducted by the same technician to ensure consistency.

### 2.4. DNA Extraction and Hydroxymethylation Measurement of LINE-1

The sperm DNA was prepared as described in our previous study [14]. Briefly, the sperm DNA was extracted by treating sperm pellets with guanidine hydrochloride and sodium citrate, followed by precipitation with ethanol. Then sperm DNA was isolated by a standard phenol/chloroform extraction method and qualified by electrophoresis on an agarose gel and visualized with ethidium bromide.

**Figure 1:**
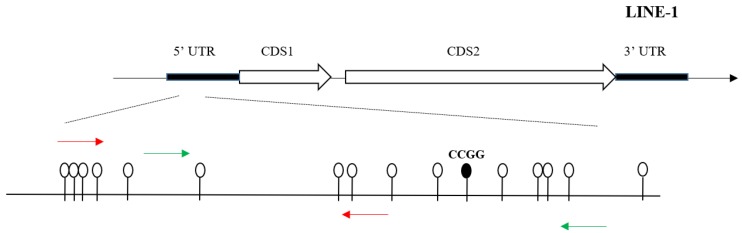
Schematic representation of location of the CCGG loci in the 5UTR of LINE-1.

DNA glucosylation and digestion was performed using the EpiMark 5-hmC and 5-mC Analysis Kit (NEB) which can be used to analyze and quantitate 5-mC and 5-hmC within a specific locus. Analyses were conducted according to the manufacturer’s protocols. Briefly, DNA was first mixed with UDP-glucose, then split into two parts which were incubated with or without T4 beta-glucosyltransferase(T4-βGT), respectively, for 16 h at 37 °C. This glucosylation was followed by restriction endonuclease digestion. Both reaction mixtures were then run in triplicate which were mock digested, digested with MspI, or digested with HpaII for at least 4 h. Samples were treated with proteinase K and incubated at 40 °C for 30 min. Proteinase K was then inactivated by incubating at 95 °C for 10 min. The fraction of glycosylated DNA and therefore protected MspI sites as well as the fraction of 5mC- and 5hmC-sensitive sites (determined using HpaII restriction) at specific gene loci in LINE-1 were quantified by qPCR using primers [34]. Schematic representation of location of the CCGG loci in the 5UTR of LINE-1 was presented in Figure 1. The rate of hydroxymethylation was calculated using the formulae in the kit according to the manufacturer’s protocols.

The red line indicates the region where enrichment of BPA-associated 5hmc was detected through high-throughput sequencing, and it is 200-bp long in LINE-1. The green line represents the location of qPCR product.

### 2.5. Statistical Analyses

Descriptive statistics on subject characteristics were tabulated by BPA occupational exposure status. All participants were further divided into four groups by urinary BPA level: BPA undetectable (lower than LOD), and lowest tertile, middle tertile and highest tertile among those with detectable BPA. We calculated means (SD) and percentiles for the distributions of 5hmC by occupational BPA exposure (yes or no) and urinary level of BPA as mentioned above. Multivariate linear regression models were used to examine the association between BPA exposure as categorized variables with hydroxymethylation of LINE-1. The following variables were adjusted as potential confounders: age, smoking, alcohol consumption and history of disease, which had been reported to be associated with DNA hydroxymethylation [35,36,37]. In addition, to examine a potential modifying effect on the association between BPA exposure and DNA hydroxymethylation of LINE-1, stratified analyses were conducted by age, smoking status, alcohol consumption and history of disease due to their known effects on 5hmC.

We ran similar linear regressions with BPA concentration (log transformed) as a continuous variable to examine its linear relationship with hydroxymethylation of LINE-1. The concentration of the BPA below the LOD was replaced with LOD/√2 as a widely accepted method. All statistical analyses were performed using SAS version 9.3 (SAS Institute Inc., Cary, NC, USA).

## 3. Results

Characteristics of exposed and unexposed male workers are presented in Table 1. The occupational BPA-exposed group had a higher proportion of males aged 30–35 years, although with a similar average age (34.1 ± 8.3 vs. 34.4 ± 9.6) as those in the unexposed group. The BPA-exposed group was less educated and less likely to be tobacco smokers or alcohol drinkers. Urine BPA concentrations are described in Table 2. The geometric mean (GM) of adjusted creatinine urine BPA concentration was significantly higher in the BPA-exposed group (158.41 μg/g Cr in the exposed group vs. 0.84 μg/g Cr in the unexposed group). The GMs of urine BPA concentration were 3.77 μg/g Cr, 33.94 μg/g Cr, and 1698.88 μg/g Cr for the lowest tertile, middle tertile, and highest tertile, respectively, for the group with detectable BPA.

The spermatic LINE-1 was more highly hydroxymethylated in the BPA-exposed group than in the unexposed group (median 12.97% vs. 9.68%; *p* < 0.05; Table 3), after adjusting for potential confounders. When dose-dependent effects were examined, the medians of 5hmC generally increased with increasing urine BPA level: 8.79%, 12.16%, 11.53%, and 13.45%, for undetected BPA and corresponding tertiles among detected BPA, respectively (Table 3). However, we did not observe a linear association between natural log transformed urine BPA levels and LINE-1 hydroxymethylation (data not shown).

We performed analyses stratified by worker’s age, smoking, alcohol consumption and history of disease. The difference in spermatic LINE-1 hydroxymethylation between the BPA-exposed group and unexposed group remained essentially unchanged across the subcategories although some estimations were attenuated slightly and became non-significant due to small sample sizes (Table 4).

## 4. Discussion

We observed that BPA exposure (based on both occupational BPA exposure status and urinary BPA concentrations divided into four groups) was associated with increased LINE-1 hydroxymethylation in sperm using individual specimens. Similar associations were also found in stratified analyses.

To our knowledge, this is the first epidemiological study on DNA hydroxymethylation in individual human sperm in relation to BPA exposure. Only two previous animal studies have examined the relationship between BPA exposure and global DNA hydroxymethylation, where maternal exposure to BPA inhibited global DNA hydroxymethylation in adult rat testis and BPA exposure led to a decrease of testicular TETs and 5hmC levels in *Gobiocypris rarus* testes [26]. 5hmC density and function appears to vary with cell type, differentiation stage and gene specific function [38]. Therefore, different cell types and genes analyzed in these studies and the disparity of species-specific transposon complement [39] may be the potential explanation for the inconsistent results. There are several potential mechanisms whereby BPA exposure could increase 5hmC. First, BPA exposure increases the generation of reactive oxygen species (ROS) [40,41], which can activate the TET protein family members that induce oxidation of 5mC into 5hmC [42]. Consistent with this model, previous studies have demonstrated that ROS-inducing chemicals, like hydroquinone and particulate matter, increase genome-wide and gene-specific 5hmC formation [43,44,45]. Another possible mechanism is through metabolic alterations that could change the rate of generation of 5hmC. BPA could influence the tricarboxylic acid cycle [46], thereby increasing the levels of α-ketoglutarate (α-KG), a known substrate for TET-catalyzed oxidation of 5mC to 5hmC [47]. In addition, one recent study has suggested that BPA might prevent initiation of the base excision repair (BER) pathway [48], which is required for demethylation of 5hmC [49].

In contrast to our previously published study [14], here we observed a more profound difference of LINE-1 hydroxymethylation than methylation (alterations of 34% for 5hmC, and 5% for 5mC) between the BPA-exposed group and unexposed group. While 5mc is associated with repression of gene expression, 5hmC is specifically present at actively transcribed genes and is mainly involved in activating gene expression [50]. Given that the 5hmC profile shows a better correlation with gene expression compared to 5mC [51], 5hmC may function as a more sensitive biomarker than 5mC in response to BPA exposure.

In addition, 5hmC content is positively correlated with the expression of retrotransposons (including LINEs, SINEs, LTRs, and satellites) during spermatogenesis [25]. Considering that the activation of LINE-1 retrotransposons is linked with male infertility [52], the increase of 5hmC level in sperm LINE-1 might have implications for understanding the mechanisms underlying BPA-induced impairment of male reproductive function. Moreover, exploring the associations between LINE-1 hydroxymethylation and BPA exposure by analyzing semen specimens rather than other tissues is more straight-forward and accurate for understanding the potential epigenetic effects of BPA-induced male infertility.

There are several limitations in our study. First, our sample size was relatively small which may have resulted in false negative findings or chance findings that limit the generalizability. Second, single-spot urine sample for assessing BPA exposure in the unexposed group may have resulted in a measurement error due to the relatively short half-life of BPA. However, the measurement error was likely non-differential, which would bias the association toward null, as previously reported [11,14]. Additionally, we did not perform “Spermatozoa purification” to separate spermatozoa from cellular contaminants since a similar protocol was used in several studies examining sperm DNA methylation in humans [53,54], which may have potential contamination by round cells. We did not collect information on BPA exposure relevant lifestyle factors, which may cause non-differential misclassification, and thus bias the association toward null. Finally, similar to other sperm studies, a proportion of participants declined to provide biological samples (urine and semen) which may have led to a selection bias.

## 5. Conclusions

In the present study, our data indicate that occupational exposure to BPA is positively associated with LINE-1 hydroxymethylation in human semen samples. Thus, our findings provide supportive evidence that 5hmC may function as a new epigenetic modification for epidemiological studies on the impact of BPA, or other environmental toxicants on male semen quality.

## Figures and Tables

**Table 1:** Characteristics of workers in the BPA-exposed and unexposed groups.

Characteristics	Exposed (*n* = 72)	Unexposed (*n* = 86)	*p*
Age (years)			
<29	23 (31.94)	32 (37.21)	0.48
30–35	21 (29.17)	18 (21.18)	
>36	28 (38.89)	36 (41.86)	
Education			
≤Middle school	21 (29.17)	20 (23.26)	0.57
High school	39 (54.17)	47 (54.65)	
≥College	12 (16.67)	19 (22.09)	
Smoking			
Yes	47 (65.28)	60 (69.77)	0.55
No	25 (34.72)	26 (30.23)	
Alcohol consumption			
Yes	16 (22.22)	22 (25.58)	0.62
No	56 (77.78)	64 (74.42)	
History of disease			
Yes	13 (18.06)	16 (18.60)	0.93
No	59 (81.94)	70 (81.40)	

**Table 2:** Urine BPA levels (μg/g creatinine) between groups.

Groups	*N*	GM (std)	Median (Q1, Q3)
By occupational exposure status			
BPA-exposed	72	158.41 (17.92)	238.78 (24.14, 2043.22)
Unexposed	86	0.84 (6.53)	LOD (LOD, 6.07)
By urine BPA level			
BPA undetectable (below LOD)	53	LOD	LOD
Low tertile (0–13.28)	35	3.77 (3.07)	4.78 (1.55, 8.19)
Middle tertile (13.28–274.82)	35	33.94 (2.13)	30.88 (22.67, 53.72)
Top tertile (274.82–)	35	1698.88 (6.74)	2158.44 (679.92, 9771.63)

**Table 3:** BPA exposure and the 5hmC rate of LINE-1.

Groups	*N*	Mean% (std)	5th%	25th%	50th%	75th%	95th%	β	*p*
By occupational exposure status									
Unexposed	86	9.68 (4.97)	2.13	6.48	8.99	11.62	20.95	Ref	-
Exposed	72	12.97 (5.07)	5.62	9.51	12.45	15.71	23.45	0.034	<0.0001
By urine BPA level									
BPA undetected	53	8.79 (4.22)	1.91	6.32	8.36	10.24	16.09	Ref	-
Low tertile	35	12.16 (5.75)	5.25	6.92	11.85	17.13	22.55	0.031	0.006
Middle tertile	35	11.53 (4.58)	5.99	8.54	10.26	15.22	21.51	0.023	0.047
Top tertile	35	13.45 (5.58)	4.71	10.23	12.71	18.46	24.15	0.048	<0.0001

Adjusted for: age, history of disease, smoking and alcohol consumption.

**Table 4:** Linear regression of occupational BPA exposure and hydroxymethylation in LINE-1 upon stratification.

Stratified Analysis	*N*	Crude		Adjusted	
		β	*p*	β	*p*
Smoking					
NO	51	0.042	0.002	0.047	0.001
YES	107	0.029	0.005	0.028	0.007
Alcohol consumption					
NO	120	0.034	<0.001	0.035	<0.001
YES	38	0.031	0.073	0.027	0.148
Age group (years)					
<30	55	0.030	0.047	0.031	0.046
30–36	39	0.017	0.334	0.013	0.473
≥36	64	0.040	<0.001	0.045	<0.001
Disease					
NO	129	0.035	<0.001	0.036	<0.001
YES	29	0.023	0.167	0.025	0.167

Smoking was not adjusted in the stratified analysis by smoking, drinking was not adjusted in the stratified analysis by alcohol consumption, age was not adjusted in the stratified analysis by age, and disease was not adjusted in the stratified analysis by disease.
